# Neuro-immune interactions in cancer: mechanisms and therapeutic prospects

**DOI:** 10.3389/fimmu.2026.1729908

**Published:** 2026-06-19

**Authors:** Jingting Liu, Chun Zhang, Chong Zhang, Jianying Pei, Yan Li

**Affiliations:** 1 Clinical Laboratory Center, Gansu Provincial Maternity and Child-Care Hospital, Lanzhou, China; 2 Clinical Laboratory, Lanzhou University of Technology Hospital, Lanzhou, China; 3 Department of Biochemistry and Molecular Biology, Medical College of Northwest Minzu University, Lanzhou, China

**Keywords:** cancer, immune systems, nervous system, neuro-immunity, tumor microenvironment

## Abstract

The interplay between the nervous and immune systems has attracted growing scientific attention due to its implications for tumor progression, immune regulation, and potential therapeutic strategies. Although their primary concentration is on inflammation and homeostasis, this basic information emphasizes the crucial role that neuro-immune interaction plays in disease states, including cancer. Neuro-immune interactions are complex and multifaceted, playing significant effects on the pathophysiology of cancer. These interactions involve both peripheral and central neural pathways, modulating immune cell activity within the tumor microenvironment, and offering promising avenues for innovative therapeutic interventions. This review synthesizes recent advancements in understanding the molecular mechanisms, regulatory pathways, and translational applications of the neuro-immune axis in cancer. We explore the intricate roles of neurotransmitters, cytokines, and neurotrophic factors in mediating this crosstalk, examining how the peripheral, central, and enteric nervous systems regulate tumor immunity. Furthermore, we explore emerging therapeutic strategies that target neural inputs, combine neuroimmune modulation with immunotherapy, and consider the impact of epigenetic regulation on the neuroimmune microenvironment. By synthesizing recent advances, this review aims to provide a comprehensive understanding of this intricate field, identify current challenges, and delineate future research directions to harness neuro-immune modulation for offering promising avenues for innovative therapeutic interventions.

## 1 Introduction

Cancer poses a significant global health challenge. With our understanding of it fundamentally changing over recent decades, we recognize that tumors are not just masses of malignant cells but are intricately integrated within the physiological networks of host. The genetic alterations are essential but not enough for initial development and progression of cancer, and complex intricate interactions. The tumor microenvironment (TME) with a highly structured ecosystem containing cancer cells was surrounded by a variety of non-malignant cell types, fuels disease progression, metastasis, and therapeutic resistance (1). The traditional view holds that the TME is mainly composed of tumor cells, immune cells, fibroblasts (2), endothelial cells, adipocytes (3), and a network of blood vessels and lymphatic vessels. With the development of the emerging interdisciplinary discipline of “cancer neuroscience” (4), increasing evidence indicates a link between neurological functions and tumorigenesis (5–8). With studies on neuro-immune crosstalk, the role of the nervous system in tumor progression has changed from a traditional passive bystander to a key active regulator (9–14). Recent advancements highlight the extensive presence and active role of neural elements-such as nerve fibers, neurotransmitters, and neurotrophic factors-in and around tumors. This challenges the long-standing belief that tumors are devoid of innervation. This neural infiltration, known as perineural invasion, is now recognized as a key route for cancer cell spread and a major factor in the immunosuppressive environment of many solid tumors. Mounting evidence highlights the extensive presence and active role of neural elements—such as nerve fibers, neurotransmitters, and neurotrophic factors—in and around tumors (15–17). This neural infiltration, known as perineural invasion, is now recognized as a key route for cancer cell spread and a major factor in the immunosuppressive environment of many solid tumors (18–20).The intricate relationship between the nervous and immune systems is a bidirectional communication where neural signals significantly impact immune responses and vice versa (14, 21–23). This insight has revolutionized our knowledge of cancer development, progression, metastasis, and the effectiveness of clinic therapies.

The nervous system spans the entire body, maintaining stability and function through the transmission of chemical and electrical signals. These signals are primarily generated and integrated by the central nervous system (CNS), while processes like stress, emotional memory, appetite, motivation, and metabolism, send feedback signals to the CNS to adjust its regulatory output (24).The relative roles of pre-existing versus newly formed neuronal networks in tumor metastasis remains under investigation. The presence of nerve fibers within tumor tissues, once thought to be irregular or absent. However, existing neurons may be sufficient to aid tumor progression, while newly formed axons can further increase the metastatic potential (25). Notably, the presence of nerve fibers within tumor tissues is now recognized as a significant feature in many solid tumors, and increased nerve density is now considered a hallmark of many solid tumors, contributing to cancer progression (9). Perineural invasion (PNI), first discovered in head and neck tumors, is the invasion of peripheral nerves by neoplastic cells (26). Since then, PNI has been investigated for its clinical relevance across various tumors, including pancreatic cancer (PDAC) (27), prostate cancer (28), colorectal cancer (CRC) (29) and breast cancer (BC) (30), which facilitating tumor spread, associated with pain, recurrence and poor outcomes. PNI encompasses both local tumor infiltration and distant metastasis, and in certain tumors, is also recognized as a form of cancer metastasis.

The complexity of neuro-immune interactions in cancer are recognized as major factor in tumor development and progression. Although the exact processes by which neurons affect immunity are still being investigated, there is mounting evidence that afferent stimulation plays a significant role in the release of sensory neuropeptides into the peripheral microenvironment (31–33). They contribute to the acquisition of aggressive tumor phenotypes, such as enhanced migration, invasion, and metastatic potential, by influencing processes like angiogenesis and metabolic reprogramming (18, 19, 34). Moreover, these interactions play a crucial role in shaping the immune landscape within the TME, often promoting an immunosuppressive state that hinders effective anti-tumor immunity and limits the success of conventional and novel immunotherapies (35–37). For instance, highly innervated tumors like PDAC and prostate cancer exhibit distinct neuro-immune profiles compared to brain tumors such as glioblastoma (GBM), where the blood-brain barrier (BBB) and unique CNS immune cells play critical roles (36, 38–41). Understanding these tissue-specific nuances is crucial for developing targeted therapeutic strategies. The emerging concept of the gut-brain-cancer axis further expands this understanding, highlighting the systemic influence of gut microbiota metabolites on neurobiology and tumorigenesis, thereby adding another layer of complexity to neuro-immune crosstalk (42).

This review aims to provide a comprehensive overview of the current understanding of neuro-immune interactions in cancer, drawing upon recent literature to delineate the molecular underpinnings, the distinct roles of different nervous system components, and the promising translational avenues for therapeutic intervention. By synthesizing findings on neurotransmitter-mediated immune regulation, cytokine-neural modulation, and neurotrophic factor signaling, we seek to elucidate the intricate mechanisms that govern this crosstalk. We will further explore how the peripheral, central, and enteric nervous systems differentially regulate tumor immunity, highlighting their unique contributions. Finally, we will discuss innovative therapeutic strategies, including those targeting neural inputs and combining neuroimmune modulation with existing immunotherapies, while also acknowledging the challenges and outlining future research directions to fully exploit the neuro-immune axis for enhanced cancer treatment. By emphasizing the extensive role of the nervous system in shaping the TME, we aim to synthesize the latest findings, predominantly from the last decade, to illuminate the critical importance of the neuro-immune axis in cancer and to identify promising avenues for improving patient outcomes.

## 2 Molecular mechanisms underlying neuro-immune interactions

The intricate dialogue between the nervous and immune systems in the context of cancer is orchestrated by a complex array of molecular mechanisms, involving a diverse repertoire of signaling molecules. These molecules act as bidirectional signaling agents, allowing neurons to modulate immune cell activity and, conversely, enabling immune cells to influence neural function and integrity within the tumor microenvironment (TME) (5, 38, 43).These include neurotransmitters, which are traditionally associated with neuronal communication but also exert profound effects on immune cells; cytokines, the classic mediators of immune responses that can also modulate neural function; and neurotrophic factors, essential for neuronal survival and growth, which have been increasingly recognized for their roles in tumor progression and immune evasion. Understanding these molecular underpinnings is crucial for deciphering how the neuro-immune axis influences cancer biology and for identifying novel therapeutic targets.

### 2.1 Neurotransmitters and neuropeptides in immune regulation

Neurotransmitters, traditionally recognized for their roles in neuronal communication, are increasingly appreciated for their profound impact on immune cell function and the overall immune landscape of the TME (5, 44). The nervous system, through its direct innervation of tumors, releases various neurotransmitters that can directly bind to receptors on cancer cells and immune cells, thereby modulating their behavior. For instance, sympathetic nerve signaling, often associated with stress responses, has been shown to predominantly promote tumor progression by rewiring the TME towards tumor-supportive phenotypes (44–47). This involves the release of neurotransmitters like norepinephrine, which can directly influence cancer cell proliferation, invasion, and metastasis, as well as modulate the activity of immune cells, potentially leading to immunosuppression (48). Conversely, the role of parasympathetic signaling, often mediated by acetylcholine, can vary depending on the cancer type, sometimes promoting and other times inhibiting tumor growth (44, 49, 50).

A specific example of neurotransmitter involvement is 5-hydroxytryptamine (5-HT), also known as serotonin. Research in non-small cell lung cancer (NSCLC) has demonstrated that nerve growth factor (NGF) drives neural infiltration into tumors, leading to significantly elevated levels of 5-HT in tumors with extensive neural infiltration (19). This nerve-secreted 5-HT was found to enhance glycolysis in NSCLC cells, a metabolic reprogramming event that is critical for tumor growth and survival. 5-HT activated the PI3K/Akt/mTOR pathway, a central signaling cascade involved in cell growth, proliferation, and metabolism, thereby promoting this metabolic shift (51). Importantly, this metabolic reprogramming contributed to the establishment of an immunosuppressive tumor microenvironment by impairing cytotoxic CD8+ T-cell activity, promoting regulatory T-cell expansion, and enhancing the secretion of immunosuppressive cytokines such as IL-10 and TGF-β (51–53). In addition, persistent activation of PI3K/Akt/mTOR signaling further facilitated immune evasion and tumor progression through chronic immunosuppressive remodeling of the TME. Zheng Y et al.’s study further highlighted that neutralizing 5-HT-mediated metabolic reprogramming could enhance the efficacy of PD-1 monoclonal antibody treatment in murine models, underscoring 5-HT’s role as a critical mediator of neuro-tumor-immune crosstalk and a potential therapeutic target (19). Beyond 5-HT, other neurotransmitters and neuropeptides also play significant roles. Lamkin DM et al’s study shows that chronic stress accelerates the development of human pre-B cell acute lymphoblastic leukemia in an orthotopic mouse model, via an indirect route controlled by β-adrenergic signaling (54).

Neuropeptides, such as Substance P, released by nociceptor neurons (55), has also been implicated in promoting immunosuppression within the TME. In breast cancer, Substance P binds to its receptor, neurokinin 1 receptor (NK1R), promoting cancer cell proliferation and lymph node metastasis (56). Through NK1R (encoded by the gene TACR1) signaling on tumor cells, Substance P mostly influences tumor-associated immune responses by increasing nuclear factor-κB (NF-κB) activity, which in turn increases cytokine production, Toll-like receptor (TLR) expression, and PD1 levels (57). Breast cancer cells triggered calcium activity in sensory neurons and released Substance P. Using 3D co-cultures and *in vivo* models, Padmanaban V et al. revealed that Substance P from neurons stimulates the growth, invasion, and metastasis of breast cancer. Additionally, tumors with higher Substance P levels showed increased lymph node metastasis. Substance P interacts with tumor tachykinin receptors (TACR1), leading to the death of TACR1^high^ cancer cells (56). In breast cancer, substance P interacts with its receptor neurokinin 1 receptor (NK1R) to stimulate cancer cell proliferation and lymph node metastasis. Apoptotic NK1R^high^ cancer cells release single-stranded RNA, which activates Toll‐like receptor 7 (TLR7) on other malignant cells and, in turn, causes a pro-metastatic gene profile leading to growth and metastasis, resulting to lower survival (56). Wang Y and colleagues also found that Substance P is highly expressed in cervical squamous cell carcinoma and enhances the proliferation and invasion of SiHa cervical cancer cells *in vitro*. This effect is linked to the activation of the ERK1/2 pathway, which increases MMP9 levels (58).

The gut microbiota also contributes to the neuro-immune axis through its metabolites, which can act as neuromodulators. The gut-brain-cancer axis, emphasizing the role of gut microbiota metabolites like short-chain fatty acids (SCFAs), tryptophan derivatives, secondary bile acids, and lipopolysaccharides (LPS) in modulating systemic processes that influence both brain health and tumorigenesis (42). Tryptophan derivatives, for example, can be precursors for neurotransmitters like serotonin, linking gut microbiota activity to neural and immune regulation, particularly in the context of neuroinflammation and brain tumors (59). This highlights a broader systemic influence on neuro-immune interactions in cancer, where distant signals can modulate local TME dynamics.

Similar neuroimmune-associated immunosuppressive remodeling has also been reported in breast and colorectal cancers, where neurotransmitter-mediated signaling contributes to immune evasion, stromal remodeling, and metastatic progression. In colorectal cancer (CRC), recent studies have identified a β2-adrenergic receptor (ADRB2)-NGF feedforward signaling circuit between sympathetic nerves and cancer-associated fibroblasts (CAFs). Norepinephrine stimulates ADRB2-dependent NGF secretion from CAFs, which subsequently enhances intratumoral sympathetic innervation and further norepinephrine accumulation, thereby establishing a self-amplifying neuro-mesenchymal interaction within the tumor microenvironment. Downstream adrenergic signaling promotes CRC progression through ADRA2A/Gi-mediated YAP activation, while CAF-derived NGF directly activates the PI3K/AKT pathway in CRC cells, enhancing tumor growth and survival. Importantly, interruption of this neuro-CAF signaling axis using TRK inhibitors attenuated YAP and AKT activation and suppressed CRC progression in preclinical models (60). PNI in CRC is further associated with an “immune cold” phenotype linked to low B2M expression, reduced CD8^+^ T cells and CD163^+^ macrophages, and decreased PD-L1, FoxP3, and LAG3 expression, establishing a direct neuro-immune coupling mechanism (61). CD3^+^, CD8^+^, and CD45RO^+^ tumor-infiltrating lymphocytes density is inversely associated with PNI in rectal adenocarcinoma, confirming clinical neuro-immune interplay (62). In BC, CAF-mediated neuroimmune remodeling similarly contributes to immunosuppression by promoting M2-like macrophage polarization and suppressing antitumor immune responses. Targeting CAF-associated signaling pathways, including CXCL12-mediated recruitment pathways, has been shown to reduce MDSC recruitment, and increasing intratumoral CD4^+^ and CD8^+^ T-cell infiltration (63). Furthermore, evidence suggests that epigenetic suppression of immune-response pathways in HER2-low TNBC exhibits distinct epigenetic suppression of immune response genes (HLA hypermethylation, downregulation of leukocyte activation and T cell signaling pathways), may further contribute to neuroimmune-associated immune escape, highlighting the complex interplay among stromal signaling, epigenetic remodeling, and tumor immunity (64).

In summary, neurotransmitters and neuropeptides are not merely signals for neuronal communication but are active participants in shaping the tumor microenvironment and modulating immune responses. Their ability to influence tumor cell metabolism, promote immunosuppression, and contribute to cancer-associated pain underscores their critical role in cancer progression. Targeting these neurotransmitter pathways represents a promising avenue for novel cancer therapies, either as standalone interventions or in combination with existing immunotherapies.

### 2.2 Cytokines in neural modulation

Cytokines, traditionally recognized as the primary communicators of the immune system, orchestrating inflammatory responses and immune cell differentiation, are increasingly understood to exert profound effects on neural function. Beyond their established roles in neuroinflammation and neuropathic processes, cytokines also directly regulate neural remodeling within the tumor microenvironment and contribute significantly to neuro-immune crosstalk in cancer (65). This bidirectional communication means that not only do immune cells respond to neural signals, but neurons and glial cells are also highly responsive to the cytokine milieu, influencing neural plasticity, pain perception, and even cognitive function in cancer patients.

Pro-inflammatory cytokines such as IL-1β, IL-6, and TNF-α can influence neuronal differentiation, axonal growth, neurotransmitter production, and neural infiltration into tumor tissues. These cytokine-mediated neural alterations may contribute to tumor progression by enhancing tumor-associated innervation and promoting neuroimmune crosstalk within the TME. IL-6 signaling has been associated with increased sympathetic nerve activity and altered neurotransmitter release, whereas TNF-α and IL-1β may modulate neuronal survival and apoptosis through NF-κB- and MAPK-dependent pathways (66). Evidence further suggests that cytokine-driven neural remodeling can facilitate perineural invasion and establish a tumor-promoting microenvironment characterized by enhanced immunosuppression and cancer cell survival. In breast and colorectal cancers, where neuroimmune interactions are increasingly recognized, cytokine-associated neural remodeling has been implicated in disease progression and metastatic dissemination (65). The IL-6/JAK2/STAT3 pathway driving pro-inflammatory cytokine elevation at the spinal cord level and its implications for tumor-infiltrating neural modulation (67). The mechanism by which neural peptides/metabolites induce monocyte-derived IL-10 and PGE2 production, promoting Treg differentiation via EP2/EP4 and IL-10 receptor signaling (68). and recent evidence that tumor-derived small extracellular vesicles reprogram sensory nerve secretory profiles to create a pro-immunosuppressive feedforward loop (69).

Evidence suggested that cytokine-driven neural remodeling may facilitate tumor-associated innervation and perineural invasion within the TME. In breast and colorectal cancers, cytokine-associated neuroimmune remodeling has been implicated in tumor progression and metastasis. Tumor-derived inflammatory cytokines (IL-6, TNF-α, IL-1β) activate neuronal receptors, modulate glial cell polarization (e.g., promoting pro-tumor M2-like astrocyte/Schwann cell phenotypes), alter neuropeptide and neurotransmitter release (substance P, CGRP, norepinephrine, acetylcholine), and ultimately reprogram immune cell subsets (MDSCs, M2 macrophages, Tregs) within the TIME to generate an immunosuppressive milieu. Tumor-associated inflammatory cytokines exert direct regulatory effects on peripheral neurons and glial cells within the tumor microenvironment. IL-6, acting via the JAK2/STAT3 pathway, modulates neuronal excitability and promotes the production of downstream cytokines including TNF-α and IL-1β, while simultaneously suppressing anti-inflammatory mediators such as TGF-β and IL-10 at the spinal level (67). This cytokine–neural feedback establishes a self-amplifying circuit: neural activation drives further cytokine production, which in turn recruits and polarizes immunosuppressive cell populations (including MDSCs, M2 macrophages, and Foxp3+ Tregs), thereby creating a TIME that favors tumor immune evasion (67, 68).

One prominent cytokine involved in neuro-immune crosstalk is transforming growth factor beta (TGF-β). Researchers have discovered that overexpressed TGF-β leads to a wide range of metabolic diseases and dysfunctions, as well as the epithelial-mesenchymal transition (EMT) and excessive ECM deposition (70, 71), which results in fibrosis, cancer, and immunological dysfunction (72). In the early phases of carcinogenesis, TGF-β suppresses tumor growth by causing apoptosis and preventing proliferation (73). Through downregulated MYC expression (74) and overexpressed cyclin-dependent kinase (CDK) inhibitors (75), TGF-β generally suppresses proliferation and encourages apoptosis. Premalignant cells can self-impose a slow-cycling state to stay dormant for long periods of time under this setting, and they develop into disseminated cancer cells. The TGF-β-regulated immunosuppressive microenvironment indirectly facilitates tumor escape (76). TGF-β signaling regulates adaptive immunity by directly expanding Treg cells, modulating the CD4+ T cell response, and controlling effector T cell function (76). It also influences the innate immune system by inhibiting NK cells and modulating the proliferation of macrophages, antigen-presenting dendritic cells, and granulocytes (77, 78). In the early stages of PDAC, TGF-β triggers apoptosis through ID1, and suppresses epithelial cell growth (79). Tumor necrosis factor alpha (TNF-α), a pro-inflammatory cytokine, has been linked to cancer-related cognitive impairment. In testicular cancer (TC) patients undergoing chemotherapy, poorer cognitive performance was associated with an increase in TNF-alpha levels (80). This finding suggests that systemic inflammatory responses, mediated by cytokines like TNF-α, can have significant neurological consequences, impacting brain function and contributing to cognitive decline (80).

Interleukin-10 (IL-10), a well-known anti-inflammatory cytokine, also plays a crucial role in neuro-immune interactions, particularly in the context of pain resolution. Chemotherapy-induced peripheral neuropathy (CIPN), a debilitating side effect of many antineoplastic agents like paclitaxel, involves neuro-immune interactions in both its development and resolution (81). Research has demonstrated that an inducible co-stimulatory molecule (ICOS) agonist antibody (ICOSaa) can alleviate paclitaxel-induced neuropathic pain in female mice. This effect was mediated by an IL-10-dependent mechanism, where ICOSaa administration increased IL-10 expression in the dorsal root ganglion (DRG), a key site for sensory neuron cell bodies. Blocking the IL-10 receptor (IL-10R) activity occluded the pain-relieving effects of ICOSaa, confirming IL-10’s critical role (81). Interleukin-6 (IL-6) is another cytokine with significant neuro-immune implications in cancer. Cancer-released small extracellular vesicles (sEVs) can elevate IL-6 expression in dorsal root ganglion (DRG), neurons, contributing to an immunosuppressive state and T-cell exhaustion (35). This suggests that IL-6, secreted by activated neurons in the TME, can act as a pro-tumorigenic cytokine by fostering an immunosuppressive environment. The unique role of IL-17B has been observed in gastroenteropancreatic neuroendocrine tumors (GEP-NETs). Digital spatial profiling revealed that duodenal gastrinomas, characterized by an immunologically “cold” microenvironment, expressed the pro-inflammatory and pro-neural factor IL-17B. Treatment of human duodenal organoids with IL-17B activated NF-κB and STAT3 signaling and induced the expression of neuroendocrine markers, suggesting that tumor-derived IL-17B can stimulate the neuroendocrine phenotype and contribute to tumorigenesis, even in the absence of robust immune cell infiltration (82).

In summary, cytokines like TGF-β, TNF-α, IL-10, IL-6, and IL-17B are pivotal molecular bridges in neuro-immune crosstalk within cancer. They mediate diverse effects, ranging from promoting tumor aggressiveness and immunosuppression to resolving neuropathic pain and influencing cognitive function. The specific context of the TME, the type of cancer, and the involved neural and immune cell populations dictate the precise role and impact of each cytokine, underscoring the complexity and therapeutic potential of modulating these cytokine-neural interactions. Furthermore, they contribute to cancer-associated pain and can even influence tumor cell phenotype and progression. The bidirectional nature of cytokine-neural communication, where immune-derived cytokines affect neural cells and neural-derived factors can influence cytokine production, underscores the complexity and importance of this molecular axis in cancer biology. Targeting specific cytokine pathways or their downstream effects holds significant promise for therapeutic interventions.

### 2.3 Neurotrophic factor signaling pathways

Neurotrophic factors are a family of proteins essential for the survival, development, and function of neurons. Traditionally studied for their roles in the nervous system, these factors and their associated signaling pathways have emerged as critical players in cancer biology, mediating complex interactions between cancer cells, nerves, and immune cells within the tumor microenvironment. Their involvement in promoting tumor growth, invasion, metastasis, and immune evasion highlights them as key components of the neuro-immune axis in cancer.

One of the most extensively studied neurotrophic factors in cancer is NGF. NGF, along with its receptor tropomyosin receptor kinase A (TrkA), is frequently overexpressed in various cancers and has been implicated in tumor progression and metastasis. A study highlighted NGF’s role in sustaining cancer cell proliferation and evading immune defense, also noting its involvement in neurogenic inflammation through the activation of immune cells and the release of pro-inflammatory cytokines (83). Zheng et al. further demonstrated that NGF drives neural infiltration in non-small cell lung cancer (NSCLC), setting the stage for 5-HT-mediated metabolic reprogramming and immunosuppression, underscoring NGF’s foundational role in establishing a pro-tumor neuro-immune environment (19).

Glial-derived neurotrophic factor (GDNF) is another crucial neurotrophic factor. GDNF, minimally expressed in the healthy adult brain but secreted by reactive CNS microglia and macrophages in response to injury, acts as a prosurvival neurotrophin (84). Recent data has revealed that GDNF is aberrantly elevated in human glioblastoma (GBM) tissues and cells (85) and promotes GBM cell invasion, migration, and proliferation through a variety of pathways (86, 87). Additionally, GBM growth is significantly slowed by decreasing GDNF and its receptor (GFRα1), indicating that GDNF may be a viable target for GBM treatment (88). In breast cancer metastasis to the leptomeninges (LM), breast cancer cells (BCCs) were found to coincide with perivascular meningeal macrophages and trigger GDNF expression (89). BCCs that express the GDNF receptor, neural cell adhesion molecule (NCAM), can transmit antiapoptotic signals, boosting their survival in nutrient-poor lymphatic microenvironments. Blocking intrathecal GDNF, eliminating GDNF in macrophages, or deleting NCAM from BCCs suppressed BC growth in the lymphatic microenvironment (89). This study underscores the role of GDNF in sustaining tumor survival in metastatic niches, highlighting the GDNF-NCAM signaling pathway as a promising therapeutic target.

Brain-derived neurotrophic factor (BDNF), with its cognate receptor tropomycin receptor kinase B (TrkB), is another neurotrophin gaining attention in cancer (90). The overexpression of TrkB and BDNF is linked to a poor cancer prognosis. This is due to their roles in metastasis formation, epithelial-mesenchymal transition (EMT), invasion, migration, and proliferation (91, 92). The activation of the TrkB pathway by BDNF in breast cancer appears linked to cell growth and metastatic behavior (93). A study has also revealed that mature BDNF and its receptor TrkB are highly expressed in human glioma cells. Mature BDNF and TrkB exhibit comparable expression profiles, showing elevated expression in tandem with the increase in glioma grades. Furthermore, high-grade samples showed enhanced co-localization of mature BDNF and TrkB in human gliomas, indicating that the mature BDNF/TrkB signaling pathway influences the prognosis and malignancy of gliomas (94). TrkB and BDNF were found to co-express in 54.4% of all cases of lung cancer, and the STAT3 proliferation pathway was found to be activated by BDNF. However, in a phase 1 clinical study, the activation of this pathway decreased when TrkB was blocked with medication (95).

**Figure 1:**
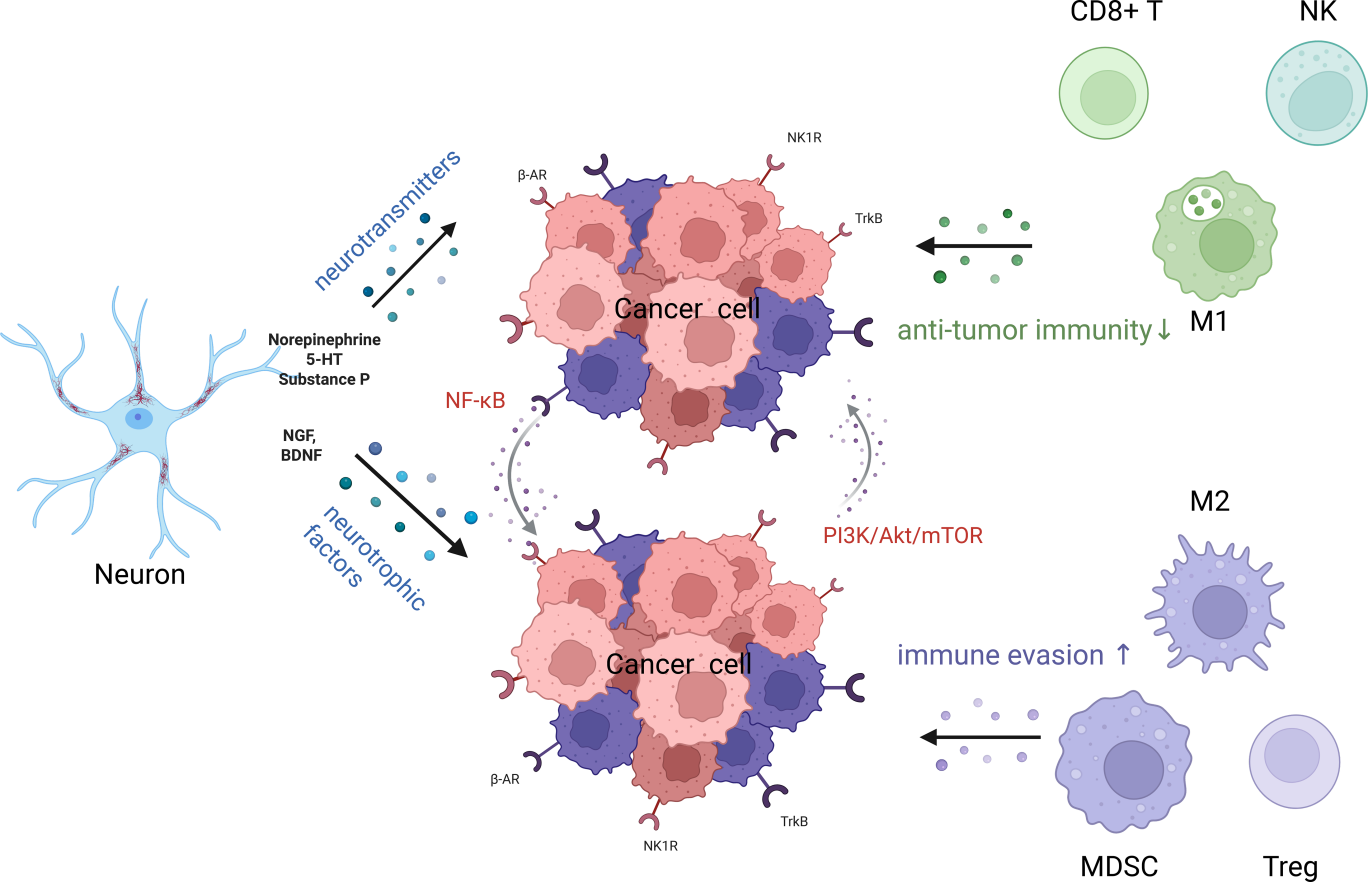
Dynamic bidirectional feedback of neuro-immune communication in cancer. Neurons release neurotransmitters (e.g., 5-HT, Substance P) and neurotrophic factors (e.g., NGF, BDNF), which bind to their cognate receptors (e.g., β-AR, NK1R) on cancer cells. This activates key oncogenic pathways (PI3K/Akt/mTOR, NF-κB), promoting cancer cell survival and the secretion of factors that induce an immunosuppressive microenvironment. Consequently, anti-tumor immunity is suppressed, characterized by inhibited CD8+ T and NK cell activity, and an expansion of immunosuppressive MDSCs and Tregs. This altered immune landscape, in turn, feeds back to further stimulate neuronal activity, creating a self-reinforcing cycle that fuels tumor growth and immune evasion.

The general concept of neurotrophic factors influencing cancer progression is further supported by the observation that in the initial or developmental stage of cancer, factors such as NGF, BDNF, and GDNF are associated with poor prognosis in various cancers by communicating with cancer cells, immune cells, and peripheral nerves within the TME. **Figure 1** summarizes the dynamic bidirectional neuro-immune feedback network within the tumor microenvironment, highlighting how neurotransmitter-mediated signaling promotes immunosuppressive remodeling and tumor progression. Research has explored preventing cancer growth by controlling the activation of these neurotrophic factors within tumors, showing promising results and offering novel attempts in cancer treatment. This collective evidence firmly establishes neurotrophic factor signaling pathways as critical regulators of the neuro-immune axis in cancer, influencing tumor growth, invasion, metastasis, and immune evasion. Their multifaceted roles make them attractive targets for developing innovative therapeutic strategies aimed at disrupting the pro-tumorigenic neuro-immune crosstalk.

## 3 Mechanisms of neural regulation of tumor immunity

**Figure 2:**
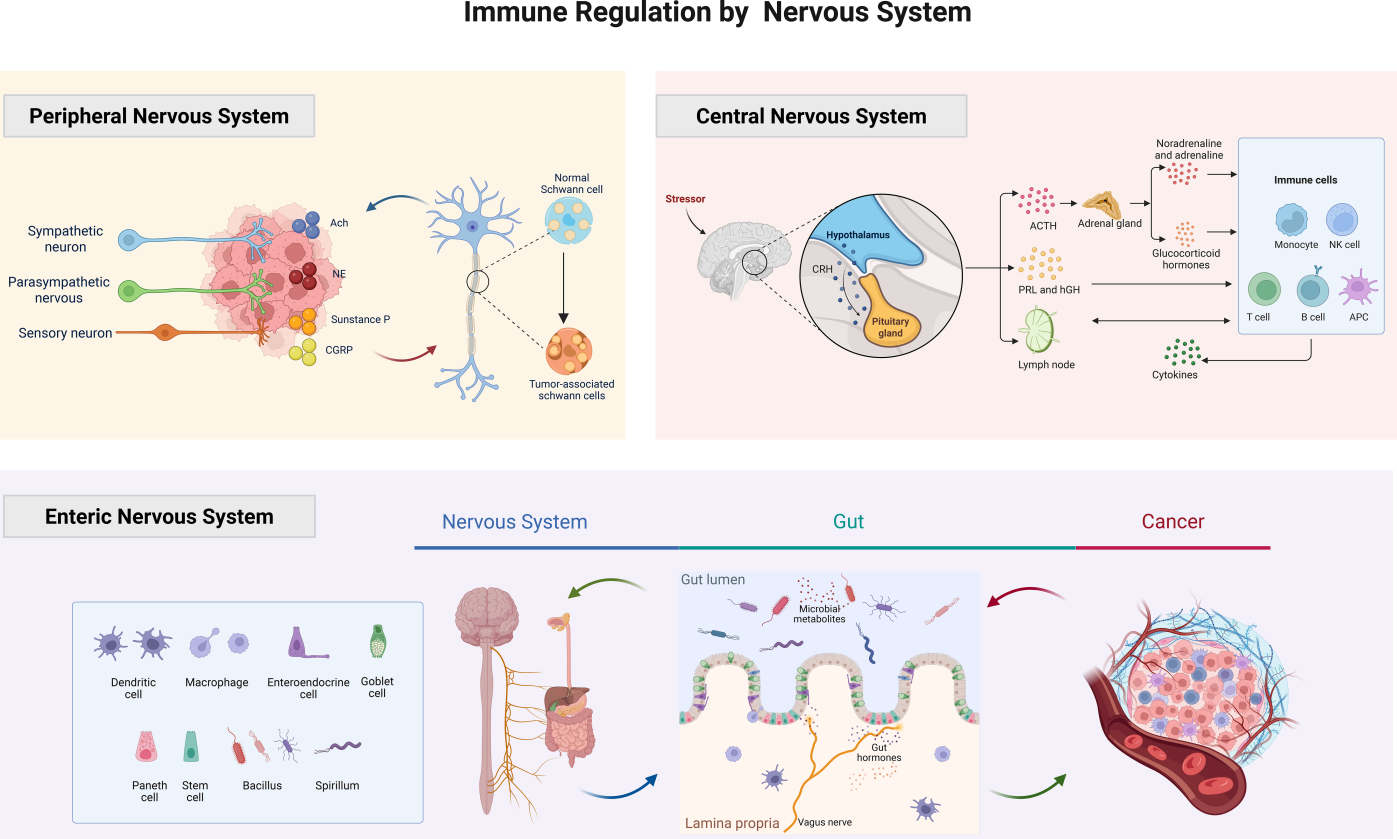
Neural regulation of the tumor immune microenvironment by peripheral, central, and enteric nervous systems. The peripheral nervous system, comprising sympathetic, parasympathetic, and sensory neurons, directly influences the tumor microenvironment via neurotransmitter release (e.g., norepinephrine, acetylcholine, Substance P, CGRP) and through tumor-supportive Schwann cells, thereby locally shaping immune cell function. Simultaneously, the central nervous system exerts systemic control through stress axes (e.g., the hypothalamic-pituitary-adrenal axis), releasing circulating factors like catecholamines that suppress cytotoxic activity in lymph nodes and the tumor bed. Furthermore, the enteric nervous system, in close communication with the gut microbiota, regulates local immune populations (e.g., dendritic cells, macrophages) and can generate systemic signals that indirectly impact distal tumor sites, collectively establishing a multi-level neuro-immune network that governs tumor progression.

The nervous system, through its diverse branches and intricate networks, exerts profound regulatory control over tumor immunity. This regulation occurs at multiple levels, involving direct innervation of tumors, systemic neuro-endocrine-immune signaling, and even the influence of distant neural systems like the enteric nervous system. **Figure 2** illustrates the multi-level regulation of tumor immunity by the peripheral, central, and enteric nervous systems through local and systemic neuroimmune signaling pathways. Understanding these distinct regulatory mechanisms is crucial for appreciating the holistic impact of the nervous system on cancer progression and for devising targeted therapeutic interventions.

### 3.1 Immune regulation by the peripheral nervous system

The peripheral nervous system (PNS), comprising sensory, sympathetic, and parasympathetic nerves, directly innervates many solid tumors, establishing a direct conduit for neuro-immune communication that significantly impacts tumor immunity (5, 44, 96). This direct innervation allows for the local release of neurotransmitters and neurotrophic factors that modulate the activity of immune cells within the tumor microenvironment (TME), often shaping an immunosuppressive landscape conducive to tumor growth and metastasis.

One of the most extensively studied aspects of PNS mediated immune regulation in cancer involves the sympathetic nervous system (SNS). The SNS postganglionic neurons emit noradrenaline which dilates airways, increases heart rate, and releases energy reserves by acting on α-adrenergic receptor (α-AR) and β-adrenergic receptor (β-AR). Adrenergic neurons mediate adrenergic signaling by releasing noradrenaline, which binds to α- or β-adrenergic receptors. Adrenergic receptor expression in cancer cells has important therapy implications, according to numerous preclinical investigations. Adrenergic agonists increase pro-tumorigenic signaling in cancer cells, while antagonists inhibit apoptosis, cell proliferation, survival, and invasion via β2-adrenergic receptor signaling (97, 98). Additionally, endothelial and immunological cells, as well as other biological components in the TME, are influenced by adrenergic signaling, which affects the mechanisms of angiogenesis, metastasis, and immune evasion (99–101). Sympathetic nerve signaling predominantly promotes tumor progression by rewiring the TME towards tumor-supportive phenotypes. These nerves interact closely with various TME components, including myeloid cells, lymphoid cells, and Schwann cells. The density of PD-L1+ tumor-associated nerves (TANs) in prostate cancer, which are often sympathetic, was found to be inversely correlated with CD8+ tumor-associated lymphocytes (TALs), suggesting that these nerves contribute to an immunosuppressive microenvironment (36). Through the β-AR, noradrenaline can also directly alter the phenotypic of T cells (31). The ADRB1 gene was upregulated in exhausted CD8^+^ T lymphocytes, which were frequently found close to sympathetic nerves. It was discovered that catecholamine exposure caused the exhaustion state in CD8^+^ T lymphocytes that expressed ADRB1. When used in conjunction with immune checkpoint blockade (ICB) to treat melanoma, β-AR antagonists that impair β1-adrenergic signaling prevented the disease from progressing to an exhausted state and enhanced CD8^+^ T cell effector activities. Additionally, combining β-blockers with ICB improved CD8^+^ T cell responses and encouraged the growth of tissue-resident memory-like T cells in a PDAC mouse model that was resistant to ICB (31).

The role of parasympathetic nervous system (PSNS) is more nuanced and can vary depending on the cancer type (44). While sympathetic activation often promotes tumor growth, parasympathetic input might have diverse effects, sometimes inhibitory and sometimes stimulatory, highlighting the complexity of autonomic nervous system regulation in cancer. Postganglionic PSNS neurons generally secrete acetylcholine (ACh), which binds to muscarinic receptors in target tissues. This action slows the heart rate, improves digestion, and stimulates glandular secretions. Despite its apparent significance, the role of PSNS signaling in tumor biology has not been thoroughly investigated. One explanation could be that parasympathetic innervation via the vagus nerve (>70% of its axons) is mostly sensory, which makes it challenging to differentiate between sensory-driven pathways and parasympathetic effects on tumors (102). Mounting evidence indicates that parasympathetic signaling contributes to carcinogenesis in hepatocellular carcinoma (HCC) (103), gastric cancer (104), and prostate cancer (105). However, in a pancreatic ductal adenocarcinoma model, the administration of the muscarinic agonist bethanechol reduced cancer stemness, decreased infiltration by CD11b^+^ myeloid cells, and decreased metastatic spread to the liver, suggesting that cholinergic signaling may limit the growth of pancreatic tumors (49). Given the widespread expression of muscarinic ACh receptors, including in immune cells, it’s unclear if these effects stem from direct stimulation of tumor cells, immune cells, or both. With the cholinergic system in T and B cells becoming a promising therapeutic target (50), more research is required to delineate the role of parasympathetic nerve signaling in tumor-immune interactions. Multiple investigations evaluated parasympathetic nerve density as an alternative biomarker for cholinergic levels in the TME. Reduced parasympathetic nerve density was directly linked to worse clinical outcomes, according to an analysis of BC (101). However, increased parasympathetic nerve fiber densities inside the TME were linked to poor survival rates in prostate cancer (106). The origin of ACh in these tumors is called into speculation by these tumor-specific effects. It’s interesting to note that ACh can be produced by liver (107), gastric (108), and lung (109) cancer cells. It has been discovered that these ACh-secreting cancer cells react to ACh, establishing an autocrine loop that encourages an ongoing proliferative stimulus (110). Notably, Ach is also produced and secreted by immunological cells, particularly T cells, indicating that cholinergic signaling may affect several cell types in the TME (111). For example, increased expression of immune checkpoint receptors was linked to parasympathetic nerve density. Reduced PD1 and PDL1 expression on tumor-infiltrating cells was observed *in vivo* when parasympathetic innervation was genetically abated. This finding is consistent with the poor prognosis observed in patients with diminished parasympathetic nerve density (101).

Sensory nerves, particularly nociceptor neurons, also play a critical role in regulating tumor immunity. The axons of peripheral sensory neurons are extensively dispersed across visceral and superficial tissues, while the dorsal root ganglia (DRG) and trigeminal ganglia are the origin of visceral sensory neurons (112). By releasing neuropeptides like substance P and calcitonin gene-related peptide (CGRP), as well as neurotrophic factors like NGF and BDNF, their terminals not only transmit mechanical, chemical, and thermal sensory signals but also regulate tumor neuro-microenvironment and encourage the malignant progression of tumors (113). Thus, these neurons become the primary link between localized malignant tumor activities and systemic physiological responses (113, 114). CGRP signaling has been directly linked to increased tumor growth by promoting cytoprotective autophagy in cancer cells (115). In head and neck tumors and oral melanoma, the production of reactive oxygen species (ROS) by cancerous cells phosphorylates JUN, which in turn triggers the transcription of NGF. Following that, NGF is released and attaches itself to its receptor, TrkA, on nociceptor neurons that innervate tumors. This promotes peri-tumoral neurogenesis, increases the transcription of calcitonin-related polypeptide-α (CALCA; the gene that codes for CGRP), and increases the secretion of CGRP. CGRP then attaches itself to malignant cells’ receptor complex of calcitonin gene-related peptide type 1 receptor (CALCRL) and receptor activity (116)modifying protein 1 (RAMP1), which promotes tumor growth and cytoprotective autophagy through the mTOR–Raptor pathway (116–118).

Beyond direct innervation, Schwann cells, which support peripheral neurons, are also key players in neuro-immune interactions. In PDAC, Schwann cells enhance the aggressiveness of cancer cells through TGF-β signaling, contributing to PNI and creating a pro-tumorigenic microenvironment (18). By releasing substances that guarantee neuronal homeostasis and response to insults, Schwann cells play a crucial role in maintaining the axonal myelin sheath (119). The invasion of lumen-lacking nerves by tumors triggers regeneration and repair programs in Schwann cells and the nerve itself, in contrast to the invasion of blood and lymphatic vessels (120, 121). The repair phenotype of activated Schwann cells facilitates the passage of cancer cells along and within the lumen-lacking nerve fibers (122).

In summary, the PNS plays a multifaceted and often pro-tumorigenic role in regulating tumor immunity, which actively regulates tumor immunity through direct innervation, neurotransmitter release, neurotrophic factor signaling, and the involvement of supporting glial cells like Schwann cells. These interactions often promote tumor growth, invasion, and immune evasion, while also contributing to debilitating symptoms. The specific effects can vary depending on the type of nerve fiber (sympathetic, parasympathetic, sensory) and the context of the tumor, necessitating a nuanced understanding for therapeutic targeting.

### 3.2 Immune regulation by the central nervous system

The CNS, comprising the brain and spinal cord, has traditionally been considered an “immune-privileged” site due to the presence of the BBB and a unique immune environment. However, this concept has been challenged by growing evidence demonstrating active immune surveillance and significant neuro-immune interactions within the CNS, particularly in the context of brain tumors like gliomas and metastatic disease. The CNS can exert both local and systemic control over immunity, influencing tumor progression and response to therapy.

Primary and metastatic CNS tumors have shown a similar phenomenon of nerve-mediated cancer growth. In comparison to the peripheral, the CNS has a remarkably high density of nerves, which make up around half of all brain cells (123), especially GBM, are among the most lethal cancers, characterized by rapid progression, infiltrative growth, therapeutic resistance and high recurrence rate, and the worst prognosis (124). Glioma progression is tightly influenced by interactions with neurons (125, 126), including invasion (127), progression (127, 128), and tumor initiation (126). Neuron-glioma connections involve both paracrine factor signaling (126, 128) and electrochemical signaling via α-amino-3-hydroxy-5-methyl-4-isoxazole propionic acid (AMPA)receptor (AMPAR)-mediated neuron-to-glioma synapses (127, 129). In line with previous findings, a study revealed that neuronal activity-regulated paracrine factors promote glioma formation (130, 131), and also reinforcing neuron-glioma connections (132). Neuroligin-3 (NLGN3) and BDNF are two important paracrine factors that are activity-regulated: NLGN3 stimulates the expression of genes encoding the AMPAR subunits GluA2 (GRIA2) and GluA4 (GRIA4), as well as TrkB2 (NTRK2), whereas BDNF-TrkB signaling enhances the trafficking of translated AMPAR subunits to the postsynaptic membrane, modulating the strength (amplitude) of postsynaptic currents. BDNF and NLGN34 both induce the formation of neuron-to-glioma synapses (132).

Except for neuronal activity-regulated paracrine factors, neurons can also directly form synapses with glioma cells to accelerate glioma progression. Glutamatergic synapses formed by neurons and glioma cells conduct signal transduction through calcium permeability AMPAR (127, 129), which is consistent with the normal synapses formed by ordinary neurons and oligodendroglial precursor cells (OPCs). Clinical models have shown that blocking glioma AMPAR can hinder the growth of glioma (127), and activating AMPA signaling pathway can accelerate the progression of glioma (129). The secretion of NLGN3 can promote the formation of neuron-glioma synapses, and the secretion of BDNF can increase the postsynaptic current intensity (132), suggesting that the regulation of synapses in neuron-glioma cells can also be enhanced by combined regulation of paracrine factors.

### 3.3 The role of the enteric nervous system

The enteric nervous system (ENS) is a highly conserved but convoluted network of neurons and glial cells, which plays a unique and increasingly recognized role in modulating systemic processes that influence both brain health and tumorigenesis. The ENS independently regulates gastrointestinal tissue dynamics and gut homeostasis, without brain or spinal cord input, thereby often referred to as the “second brain” (133). As an enormous division of the autonomic nervous system, the ENS rivals the spinal cord in complexity. Located in the body’s largest sensory organ, it collaborates with the immune system, intestinal epithelia, enteroendocrine system, and intestinal microbiome to facilitate the absorption of nutrients, water, and electrolytes while also blocking access to potentially dangerous substances found in the lumen (134).

The intricate connection with the gut microbiota forms the basis of the emerging concept of the gut-brain-cancer axis, which represents a novel and complex interplay between the gut microbiota, neurobiology, and cancer progression (42). While not directly innervating tumors in the same way as the peripheral somatic or autonomic nervous systems, the ENS, through its influence on gut function and microbiota composition, can indirectly but profoundly impact neuro-immune interactions relevant to cancer. A study explored the gut-brain-cancer axis, focusing on how gut microbiota metabolites that influence both brain health and tumorigenesis. Gut dysbiosis can alter metabolite profiles, leading to neuroinflammation and immune dysregulation that can impact tumor development and progression within the gastrointestinal tract (42).

PDAC is a prime example where the ENS and its associated neural components play a significant role in tumor progression and immune evasion. PDAC is characterized by an abundant desmoplastic stroma and pronounced perineural invasion, making nerve-cancer interactions a defining feature of this highly aggressive cancer (46, 135). Demir et al. extensively reviewed nerve-cancer interactions in PDAC, highlighting that intratumoral nerves are a rich source of neurotrophic factors (NGF, GDNF, artemin), neuronal chemokines (fractalkine), and autonomic neurotransmitters (norepinephrine) (46). These factors enhance the invasiveness of PDAC cells, trigger neural invasion (NI), and activate pro-survival signaling pathways. Conversely, PDAC cells provide trophic agents to intrapancreatic nerves, leading to remarkable neuroplasticity, augmented local neuro-surveillance, neural sensitization, and neuropathic pain. This bidirectional trophic support creates a vicious cycle that fuels tumor progression and nerve involvement. The strong correlation of NI with PDAC-associated desmoplasia suggests a triangular relationship between nerves, PDAC cells, and other stromal partners like myofibroblasts and pancreatic stellate cells, which generate tumor desmoplasia.

The ENS’s influence on PDAC extends to modulating the tumor immune microenvironment (TIME). Luo et al. provided a comprehensive review of TIME-based therapies in PDAC, emphasizing that interactions among cancer cells, immune cells, cancer-associated fibroblasts (CAFs), and extracellular matrix (ECM) components drive PDAC towards a more immunosuppressive direction (37). Xu et al. specifically discussed the regulatory mechanisms and crosstalk among stromal cells and their factors within the PDAC microenvironment, including the dynamic changes between tumor cells and surrounding nerves, immune, and stromal cells (135). They highlighted the role of neural factors like NGF and BDNF, and the sympathetic and parasympathetic nervous systems in regulating tumor cell growth, migration, and invasion, all of which contribute to the immunosuppressive TIME.

Gastroenteropancreatic neuroendocrine tumors (GEP-NETs), which arise from neuroendocrine cells in the digestive system, also exhibit unique neuro-immune interactions influenced by the ENS. Duan et al. utilized digital spatial profiling to reveal tissue-specific neuro-immune signatures in GEP-NETs (82). They found that gastrin-secreting and non-functional pancreatic NETs showed a higher abundance of immune cell markers and immune infiltrate compared with duodenal gastrinomas. Interestingly, duodenal gastrinomas were characterized by an immunologically “cold” microenvironment but expressed the pro-inflammatory and pro-neural factor IL-17B. Treatment of human duodenal organoids with IL-17B activated NF-κB and STAT3 signaling and induced neuroendocrine markers, demonstrating a cell-autonomous expression of immune and pro-inflammatory factors by tumor cells that stimulate the neuroendocrine phenotype.

## 4 Therapeutic strategies targeting the nervous system

**Figure 3:**
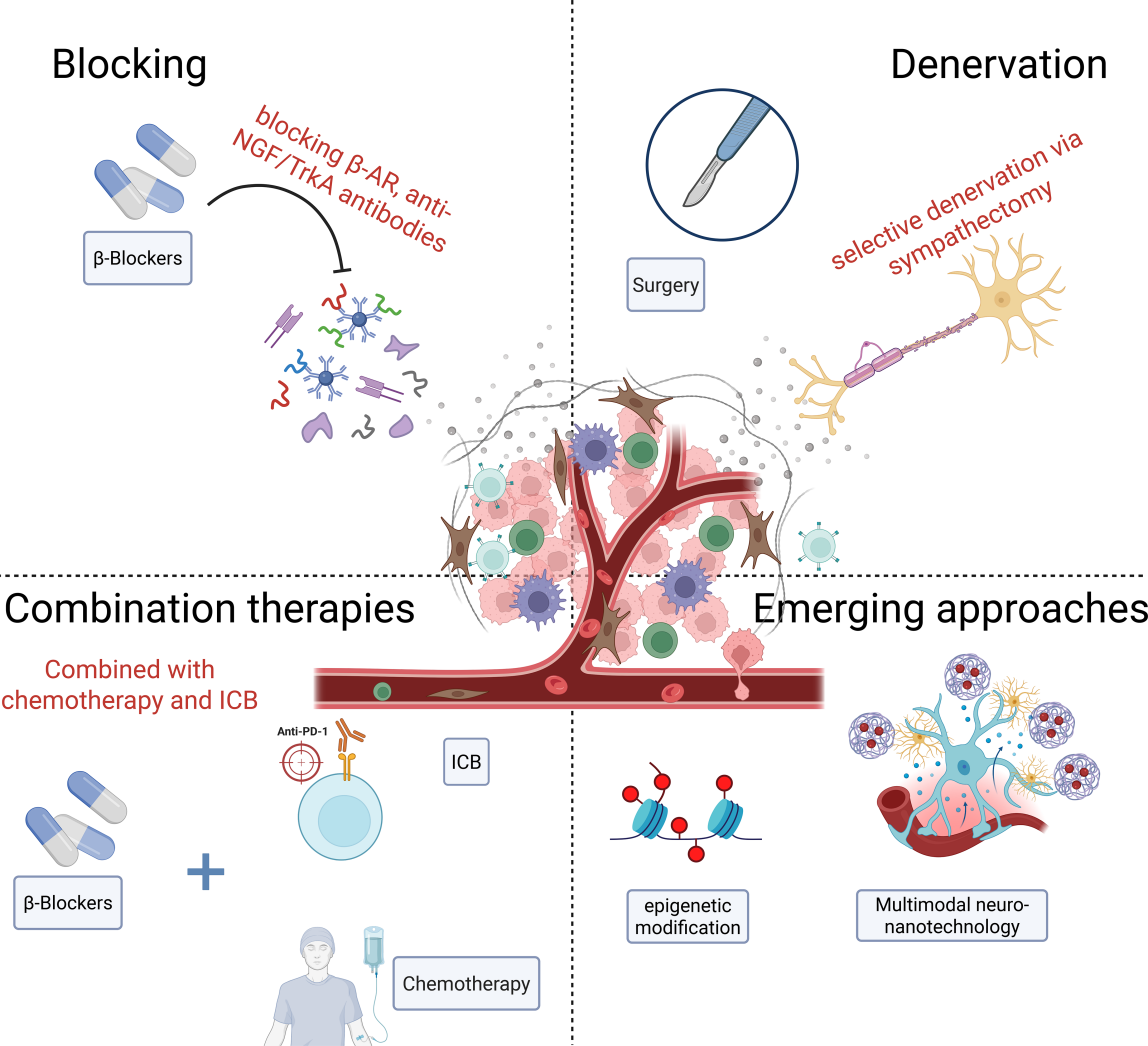
Therapeutic strategies targeting the neuro-immune axis in cancer. Current approaches encompass a multi-pronged strategy to disrupt pro-tumorigenic neuro-immune signaling, including: (1) direct blockade of neural inputs via pharmacological inhibition (e.g., β-blockers, anti-NGF/TrkA antibodies) or surgical/chemical denervation (e.g., sympathectomy); (2) rational combination therapies that integrate these neuro-modulatory agents with conventional chemotherapy and immune checkpoint blockade (ICB, e.g., anti-PD-1) to overcome immunosuppression and enhance anti-tumor immunity; and (3) emerging advanced interventions such as epigenetic modifiers to reverse T-cell exhaustion and for spatiotemporally controlled, precision drug delivery within the tumor microenvironment.

The growing understanding of neuro-immune interactions in cancer has opened promising avenues for innovative therapeutic interventions. By targeting specific components of the neuro-immune axis, researchers aim to disrupt pro-tumorigenic signaling, enhance anti-tumor immunity, and alleviate cancer-related symptoms. These translational applications range from directly modulating neural inputs to combining neuroimmune strategies with existing immunotherapies and exploring broader systemic influences. To bridge the gap between basic mechanistic discoveries and clinical implementation, it is essential to delineate the translational maturity of current neuro-immune interventions. While advanced experimental modalities offer unprecedented mechanistic precision in preclinical rodent models, their clinical application remains constrained by delivery vectors, safety profiles, and ethical boundaries. Conversely, the repurposing of established neuropsychiatric agents, particularly β-blockers, represents the most clinically advanced strategy to date. **Figure 3** summarizes current and emerging therapeutic strategies targeting neuroimmune signaling in cancer. To provide a clear and scannable overview for readers, we have structured and categorized these existing therapeutic strategies based on their current translational stages in **Table 1**.

**Table 1:** Translational landscape of neuro-immune therapeutic strategies in cancer.

Therapeutic strategy	Primary target/mechanism	Translational stage	Current status & limitations	Key evidence/clinical trials	Reference
β-blockers (Propranolol + Pembrolizumab)	No significant changes in treatment-associated biomarkers, an increase in IFN-γ, and a decrease in IL-6 in responders.	Clinical (Phase I/II)	Drug repurposing with known safety profiles; main challenge lies in identifying patient cohorts highly responsive to sympathetic stress.	NCT03384836	
Perioperative COX-2 and β-adrenergic blockade (Propranolol); Preoperative β-blockade with propranolol	Sympathetic signaling inhibition; dampens stress-induced immunosuppression.	Clinical (Phase II)	Demonstrated improved immune biomarkers in breast cancer patients; further large-scale trials are needed to confirm survival benefits.	NCT00502684; ACTRN12615000889550	(136, 137);
Perioperative COX-2 +β-adrenergic blockade	Combined inhibition of inflammatory and neural signaling pathways.	Clinical (Phase II)	Proven reduction in pro-tumor monocyte infiltration and enhancement of NK cell activity; requires optimal dosing protocols for translation.	NCT00888797	(138)
Cannabinoid receptor modulators	CB1/CB2 receptors on neural and immune cells; modulates neuro-inflammation and suppresses T-cell exhaustion.	Clinical (Phase I/II)	Exhibits dual anti-tumor and palliative (pain relief) efficacy; however, the psychoactive properties of certain compounds require precise formulation.	NCT01812603; NCT01812616	(139)
Perioperative lidocaine administration	Inhibition of neutrophil extracellular traps (NETs) and neural-driven metastasis markers.	Clinical (Prospective, Randomized)	Decrease in neutrophil extracellular trapping and MMP3 following administration of lidocaine; Safety confirmed as an adjuvant; future long-term follow-up is necessary to validate its impact on recurrence-free survival.	NCT02839668	(140)
Surgical or pharmacological denervation of the stomach	Cholinergic nerve fibers; blocks acetylcholine (ACh) release to inhibit vagal-mediated tumor promotion.	Preclinical	Proven highly effective in murine models of gastric and prostate cancer; human translation is currently limited to small-scale pilot feasibility studies.	Denervation and cholinergic antagonism, in combination with other therapies, could represent a viable approach for the treatment of gastric cancer and possibly other solid malignancies.	(104)
β--blocker (Propranolol) + anti-CTLA-4	Targets β-adrenergic receptors; enhances T cell infiltration and reduces MDSC-mediated immunosuppression in TME.	Preclinical	Preclinical models of fibrosarcoma and colorectal cancer	Demonstrates significant efficacy in murine models; further studies are required to confirm safety and optimal sequencing in humans.	(141)

### 4.1 Resource identification initiative

Directly targeting neural inputs to tumors represents a burgeoning area of cancer therapy, building on the evidence that nerves often promote tumor growth, invasion, and immune evasion. The strategies can involve surgical denervation, pharmacological modulation of neural receptors, or interventions that disrupt neurotrophic factor signaling. Studies have shown that by triggering the sympathetic signaling cascade, tumor-derived LIF and Gal3 promote the development of MDSCs (142). This neurological route is blocked by selective denervation via splenic sympathectomy, which directly improves MDSCs’ immunosuppressive capabilities (142). Benzaquen et al. highlighted that denervation has shown promise *in vitro* and in animal models, particularly for cancers like prostate cancer and PDAC, which are richly innervated. They noted that surgical or chemical sympathectomy has been shown to prevent the development of prostate cancer, suggesting that disrupting sympathetic nerve input can have therapeutic effects (143). Li et al. also mentioned that denervation of the tumor has been reported to enhance cancer metastasis in some contexts, indicating a complex role that requires careful consideration of the specific neural pathways involved and the tumor type (34). However, the potential for denervation to reduce tumor growth and spread, especially in highly innervated tumors, remains a compelling area of research.

Pharmacological interventions targeting neurotransmitter receptors or their synthesis pathways are another key strategy. Given the role of sympathetic signaling in promoting tumor progression, beta-blockers, which antagonize β-adrenergic receptors, have been investigated. The process of tumor metastasis can be effectively inhibited by propranolol, a non-selective β-adrenergic receptor antagonist, which can selectively prevent the activation of noradren-ergic cAMP/PKA signal transduction cascades (144). Study showed that patients using β-blockers had a higher survival rate than those not on β-blockers in retrospective evaluations of patients with non-small cell lung cancer treated with ICB (145). A similar result has been observed in metastatic melanoma (146). Mellgard G er al. also indicated that β-blocker enhance the clinical activity of ICB and had significant association with OS in urothelial carcinoma subgroup, compared with non-β-blocker group (147). These findings highlight thatβ -blocker may play a role in influencing the immune activity of ICB. β-blocker are safe, low-cost, and widely available agents for serving as an adjunct to ICB therapy.

### 4.2 Combining neuroimmune modulation with immunotherapy

The integration of neuroimmune modulation with existing immunotherapies, particularly ICB, holds immense promise for overcoming resistance mechanisms and enhancing therapeutic efficacy. Many neuro-immune interactions contribute to an immunosuppressive TME, which can limit the effectiveness of immunotherapies. By disrupting these pro-tumor neuro-immune pathways, it may be possible to “reprogram” the TME to be more responsive to immune-based treatments.

One clear example comes from NSCLC, where neural infiltration driven by NGF leads to 5-HT secretion, which in turn enhances tumor glycolysis and exacerbates immunosuppression, thereby impacting immunotherapy efficacy (19). The study found that neutralizing this 5-HT-mediated metabolic reprogramming significantly enhanced the efficacy of PD-1 monoclonal antibody treatment. This provides a direct rationale for combining strategies that target neural inputs (e.g., 5-HT signaling) with ICB to improve patient outcomes. Similarly, in prostate cancer, the prevalence of PD-L1 expression in TANs and its inverse correlation with CD8^+^ TALs suggest that neuro-immunological interactions contribute to an immune-suppressive microenvironment (36). This finding implies that combinatorial treatment regimens targeting neural PD-L1 and TALs, perhaps by modulating neural activity or blocking PD-L1 on nerves, could be warranted in future clinical applications of anti-PD-L1/PD-1 immunotherapy. Propranolol has been shown to be effective by modifying the immune milieu and inhibiting the establishment of tumor vasculature. In particular, it increases T-cell infiltration and decreases MDSC recruitment, which increases susceptibility to therapy that targets cytotoxic T-lymphocyte-associated protein 4 (CTLA-4) (141). This supports combination therapy with checkpoint inhibitors by indicating that propranolol blocks the adrenergic receptor signaling pathway to provide an immunomodulatory impact.

Recently, multimodal neuro-nanotechnology has emerged as a promising strategy for precise modulation of the neuro-immune axis in cancer. This approach integrates nanotechnology-based delivery systems with neural regulation and immunotherapeutic interventions to achieve spatiotemporally controlled modulation of the tumor microenvironment (148). Nanoparticles can be engineered to deliver immunomodulatory agents, neuro-modulatory molecules, or gene-editing cargos directly to tumor-associated neural and immune compartments, thereby enhancing antitumor immune responses while minimizing systemic toxicity (149). In glioblastoma models, neuro-nanotechnology platforms such as spherical nucleic acids and polymer-based nanoparticles have demonstrated the ability to improve immune activation, promote antigen presentation, and overcome local immunosuppression (150, 151). Multimodal neuro-nanotechnology offers an advanced approach to integrate precision immunotherapies with existing systemic immunotherapies, particularly for challenging cancers like GBM (19). This strategy aims to train the immune system to efficiently identify and eradicate cancer cells while minimizing multi-mechanistic immune suppression (152). The controlled, spatiotemporal delivery of structurally defined nanotherapeutics into the TME, for instance, can activate antigen-presenting cells and prime antigen-specific T cells. The efficacy of such nanotechnology-based immunotherapies can be enhanced when integrated with emerging precision surgical techniques and when combined with systemic immunotherapies, especially inhibitors of immune-mediated checkpoints and immunosuppressive adenosine signaling (148). This represents a sophisticated integration of neuro-technology, nanotechnology, and immunotherapy to overcome treatment resistance (153). Despite its considerable therapeutic potential, several major challenges currently limit the clinical translation of multimodal neuro-nanotechnology. First, achieving efficient and selective delivery across biological barriers, particularly the blood–brain barrier, remains technically difficult (148, 154). Second, nanoparticle biodistribution, long-term biosafety, and immune-related toxicity require further evaluation in human studies (155). In addition, the complexity of integrating neural modulation with immunotherapy introduces substantial challenges in treatment standardization, dosing optimization, and reproducibility across tumor types. Therefore, although multimodal neuro-nanotechnology represents a highly innovative direction for cancer therapy, most applications remain in the preclinical stage and require further validation before widespread clinical implementation.

Future therapeutic strategies may focus on integrating neuro-modulatory approaches with precision immunotherapy, epigenetic regulation, and nanotechnology-based delivery systems to achieve spatiotemporally controlled remodeling of the tumor neuroimmune microenvironment. Emerging approaches targeting neural innervation, neurotransmitter signaling, and neuroimmune metabolic reprogramming may further improve therapeutic responsiveness and overcome immunosuppressive resistance mechanisms.

### 4.3 Epigenetic regulation and impact on the neuroimmune microenvironment

Epigenetic mechanisms, including DNA methylation, histone modifications, and non-coding RNA regulation, play a fundamental role in controlling gene expression without altering the underlying DNA sequence. In the context of cancer, epigenetic dysregulation is a well-established driver of tumorigenesis and progression. Increasing studies are revealing that epigenetic modifications also profoundly influence neuro-immune interactions within the TME, impacting both neural plasticity and immune cell function, thereby offering novel targets for therapeutic intervention. The intricate interplay between epigenetic regulation and the neuroimmune microenvironment is multifaceted. Epigenetic changes can directly affect the expression of genes involved in neural development, neurotransmitter synthesis, and neurotrophic factor signaling within the TME. Conversely, signals from the nervous system and immune cells can induce epigenetic modifications in cancer cells and other stromal components, further shaping the tumor landscape. For instance, the neurotrophic factors and neurotransmitters discussed in earlier sections can activate signaling pathways that lead to changes in chromatin structure and gene expression, influencing the phenotype and function of cells in the TME. Neurotransmitters and neuroendocrine mediators, including norepinephrine, dopamine, and acetylcholine, can activate intracellular signaling pathways in immune cells through receptor-dependent mechanisms, thereby inducing epigenetic alterations such as DNA methylation, histone modification, and non-coding RNA regulation. These epigenetic changes subsequently reshape transcriptional programs that govern immune cell activation, differentiation, cytokine production, and immune exhaustion (156, 157).

Previous research has indicated that epigenetic modifications are crucial in controlling the development and adaptability of CD8^+^T cells (158, 159). Through changes in histone modification states that either stimulate or inhibit gene transcription, these mechanisms synchronize effector-associated transcriptional programs with dynamic epigenetic remodeling (159, 160). Targeting inhibitory transcription factors (TFs) and epigenetic regulators can improve T cell activity. According to Ghoneim HE et al.’s study, CD8^+^ T cells with Dnmt3a deficiency showed improved effector functions and increased sensitivity to PD-1 inhibition because they lacked the gene-specific methylation program found in wild-type T cells (161). A similar mechanism has been observed in patients with chronic lymphocytic leukemia, TET2-deficient CD19 CAR T cells showed a changed epigenetic landscape, rerouting their development trajectory toward persistent anticancer activity and causing remission (162). In melanoma, Liu G et al. has recently discovered PR domain zinc finger protein 12 (Prdm12) as a major epigenetic modulator of CD8^+^ T cell antitumor immunity using an *in vivo* CRISPR screen with an MKO library (163). Prdm12 regulates T cell activation, differentiation dynamics, migratory capacity, and cytotoxic activity, according to the study. This study provides crucial data for developing immunotherapy or neuro-immunotherapy techniques through Prdm12 targeting and demonstrates the important regulatory role of Prdm12 in T cell effector differentiation (163).The above findings highlight how epigenetic modification can be used therapeutically to overcome ICB resistance and address the drawbacks of CAR T cell treatments, specifically their functional persistence and durability. Additionally, in endogenous or adoptively transferred T cell treatments, epigenetic patterns could function as prognostic biomarkers for clinical outcomes.

In addition, recent neuroimmune studies indicate that neural stress-related signaling may induce persistent epigenetic reprogramming of immune cells, generating long-lasting inflammatory or immunosuppressive phenotypes. Histone modifications such as H3K27 acetylation and H3K4 methylation have been implicated in neuroinflammation-associated immune memory and microglial activation, highlighting the dynamic role of epigenetic mechanisms in mediating neural regulation of immune responses (164). These findings suggest that epigenetic regulation is not merely an independent modulatory layer but a central mechanistic mediator through which neural signals reshape immune cell behavior in cancer. Targeting these neuro-epigenetic pathways may therefore provide novel opportunities for precision modulation of the tumor neuroimmune microenvironment and enhancement of cancer immunotherapy. While the provided literature does not explicitly delve into the direct epigenetic regulation of neuro-immune interactions in cancer, the pervasive nature of epigenetic control in both neural and immune systems strongly implies its significant, albeit underexplored, role. Future research directions could explicitly investigate how neural signals (e.g., neurotransmitters, neurotrophic factors) induce epigenetic changes in immune cells within the TME, and how epigenetic alterations in cancer cells or stromal cells influence their responsiveness to neural modulation. Understanding these epigenetic layers of regulation could uncover novel therapeutic targets, allowing for the development of epigenetic drugs that specifically reprogram the neuroimmune microenvironment to favor anti-tumor immunity. This represents a critical research gap and a promising frontier for advancing our understanding and treatment of cancer. Although current evidence directly linking epigenetic regulation to neuroimmune interactions in cancer remains limited, emerging studies suggest that neuro-epigenetic remodeling may represent an important future direction for understanding tumor-associated neuroimmune regulation and therapeutic resistance.

## 5 Discussion: challenges and opportunities

The rapidly evolving field of neuro-immune interactions in cancer has unveiled a complex and dynamic landscape, offering profound insights into tumor biology and promising avenues for therapeutic innovation. However, translating this intricate understanding into effective clinical strategies presents a multitude of challenges that necessitate focused future research. The previous studies, while illuminating, also highlight significant gaps in our knowledge and methodological limitations that must be addressed to fully harness the potential of the neuro-immune axis in oncology.

One of the primary challenges lies in the heterogeneity and complexity of neuro-immune interactions across different cancer types and anatomical locations (82, 165). As highlighted, the role of the nervous system can vary significantly, with sympathetic signaling often promoting tumor progression (45, 166), while parasympathetic roles are more variable (44, 166, 167), and sensory nerve activity can have contradictory effects depending on tumor stage and aggressiveness (165, 168). For instance, PDAC exhibits distinct neuro-immune interactions involving Schwann cells and neurotrophic factors (18, 46), while GBM presents unique challenges due to neuroinflammation and the BBB (39). Future research must move beyond generalized observations to Future research needs to systematically map these interactions across a broader spectrum of cancers, conducting tissue-specific and cancer-type-specific analyses to delineate the precise neural circuits, molecular mediators, and immune cell populations involved in each context.

A second challenge is the bidirectional and dynamic characteristic of neuro-immune interaction, which makes it difficult to pinpoint primary drivers and effective intervention points. Cancer cells influence nerve growth, and nerves influence cancer cells and immune cells, creating complex feedback loops (34, 169). For example, tumor-released small sEVs attract nociceptive nerves, which then promote immunosuppression (35). Methodological limitations also pose a significant hurdle. While animal models provide valuable insights, translating findings to humans is complex. The intricate innervation patterns and neuro-immune circuits in humans are difficult to fully recapitulate in preclinical models. Mafe AN et al. mentioned standardized multi-omics approaches and bi-directional research frameworks integrating microbiome, neuroscience, and oncology, particularly in human longitudinal studies, to overcome current limitations in understanding the gut-brain-cancer connection (42). Future studies need to employ longitudinal and dynamic models that can capture these evolving interactions over time, from early tumorigenesis to metastasis and treatment response. This could involve advanced *in vivo* imaging techniques and sophisticated *ex vivo* co-culture systems that mimic the complexity of the TME.

Thirdly, the translation of promising preclinical findings into effective clinical therapies faces significant hurdles. While strategies like targeting neural inputs (e.g., denervation, β-blockers, neurotrophic factor inhibitors) and combining neuroimmune modulation with immunotherapy show promise (19, 89), clinical trials are needed to validate their efficacy and safety. The systemic nature of some neuro-immune interventions (e.g., targeting gut microbiota metabolites) also requires careful consideration of potential off-target effects (42). Future efforts should focus on developing highly specific and localized neuro-modulatory agents to minimize systemic toxicity, potentially leveraging advanced delivery systems like multimodal neuro-nanotechnology (148, 153).

In summary, the field of neuro-immune interactions in cancer is rapidly advancing, moving from descriptive observations to mechanistic insights. The challenges ahead involve overcoming tumor and patient heterogeneity, refining methodological approaches, elucidating dynamic feedback loops, improving drug delivery to neural sites, and effectively integrating neuroimmune modulation with existing cancer therapies. Future directions will focus on precision medicine approaches, leveraging advanced multi-omics and spatial technologies, developing novel nanotechnologies, and designing innovative combination therapies that target specific neuro-immune pathways to both combat cancer progression and alleviate patient suffering. The goal is to translate this profound understanding into tangible clinical benefits, offering new hope for patients facing this complex disease.

## 6 Conclusion

In conclusion, the neuro-immune axis has emerged as a fundamental regulator of cancer progression and therapeutic responsiveness. Evidence suggests that tumor-associated neural infiltration and neurotransmitter release actively reshape the tumor microenvironment by promoting immunosuppressive signaling pathways, including IL-10- and TGF-β-mediated immune regulation. This neuroimmune remodeling suppresses antitumor immune responses and facilitates tumor growth, metastasis, and therapeutic resistance, particularly in breast and colorectal cancers. Looking forward, integrating neuroscience, immunology, and oncology with emerging technologies such as single-cell sequencing, spatial transcriptomics, and multimodal neuro-nanotechnology may further elucidate the spatial and molecular architecture of neuroimmune interactions. Deeper investigation of neuro-epigenetic regulation may provide novel opportunities for precision therapeutic targeting and tumor microenvironment reprogramming.
